# Plasma Levels of Macrophage Migration Inhibitory Factor and d-Dopachrome Tautomerase Show a Highly Specific Profile in Early Life

**DOI:** 10.3389/fimmu.2017.00026

**Published:** 2017-01-25

**Authors:** Thierry Roger, Luregn J. Schlapbach, Anina Schneider, Manuela Weier, Sven Wellmann, Patrick Marquis, David Vermijlen, Fred C. G. J. Sweep, Lin Leng, Richard Bucala, Thierry Calandra, Eric Giannoni

**Affiliations:** ^1^Infectious Diseases Service, Lausanne University Hospital, Lausanne, Switzerland; ^2^Paediatric Intensive Care Unit, Lady Cilento Children’s Hospital, Children’s Health Queensland, South Brisbane, QLD, Australia; ^3^Paediatric Critical Care Research Group, Mater Research Institute, University of Queensland, Brisbane, QLD, Australia; ^4^Department of Pediatrics, Bern University Hospital, University of Bern, Bern, Switzerland; ^5^Service of Neonatology, Lausanne University Hospital, Lausanne, Switzerland; ^6^Department of Neonatology, University of Basel Children’s Hospital (UKBB), Basel, Switzerland; ^7^Department of Biopharmacy, Institute for Medical Immunology, Université Libre de Bruxelles (ULB), Brussels, Belgium; ^8^Department of Laboratory Medicine, Radboud University Medical Centre, Nijmegen, Netherlands; ^9^Department of Medicine, Yale University, New Haven, CT, USA

**Keywords:** macrophage migration inhibitory factor, d-dopachrome tautomerase, innate immunity, fetus, neonate, healthy adult, bronchopulmonary dysplasia, sepsis

## Abstract

Macrophage migration inhibitory factor (MIF) is a pleiotropic, constitutively expressed, pro-inflammatory cytokine and an important regulator of immune responses. d-dopachrome tautomerase (DDT), a newly described member of the MIF protein superfamily, shares sequence homology and biological activities with MIF. We recently reported that high expression levels of MIF sustain innate immune responses in newborns. Here, we elected to further characterize age-dependent MIF expression and to define whether DDT shares a similar expression profile with MIF. Therefore, we delineated the circulating concentrations of MIF and DDT throughout life using a large cohort of 307 subjects including fetuses, newborns, infants, children, and adults. Compared to levels measured in healthy adults (median: 5.7 ng/ml for MIF and 16.8 ng/ml for DDT), MIF and DDT plasma concentrations were higher in fetuses (median: 48.9 and 29.6 ng/ml), increased further at birth (median: 82.6 and 52.0 ng/ml), reached strikingly elevated levels on postnatal day 4 (median: 109.5 and 121.6 ng/ml), and decreased to adult levels during the first months of life. A strong correlation was observed between MIF and DDT concentrations in all age groups (*R* = 0.91, *P* < 0.0001). MIF and DDT levels correlated with concentrations of vascular endothelial growth factor, a protein upregulated under low oxygen tension and implicated in vascular and lung development (*R* = 0.70, *P* < 0.0001 for MIF and *R* = 0.65, *P* < 0.0001 for DDT). In very preterm infants, lower levels of MIF and DDT on postnatal day 6 were associated with an increased risk of developing bronchopulmonary dysplasia and late-onset neonatal sepsis. Thus, MIF and DDT plasma levels show a highly specific developmental profile in early life, supporting an important role for these cytokines during the neonatal period.

## Introduction

Macrophage migration inhibitory factor (MIF) is a pleiotropic cytokine that is constitutively expressed by virtually all cell types and tissues and circulates in the bloodstream of healthy adults at around 2–10 ng/ml (1). MIF promotes inflammatory, proliferative, and angiogenic biological activities and is involved in the pathogenesis of infectious, inflammatory, autoimmune, and neoplastic diseases (1, 2). Accordingly, MIF blood levels are increased in patients with arthritis (3), systemic lupus erythematosus (4), psoriasis (5), atopic dermatitis (6), ulcerative colitis (7), asthma (8), and cancer (9). MIF levels are also increased in the circulation of septic patients and correlate with dysregulated pituitary and adrenal function, inflammatory response, severity scores, and disease outcome (10–13). Functional polymorphisms of the *MIF* gene affecting MIF expression levels have been associated with susceptibility to or severity of autoimmune, infectious, and oncologic diseases (14–17). Altogether, MIF is considered as a potential biomarker/genetic marker and an attractive target for immunomodulating therapies for a number of diseases (14, 15, 18–24).

Macrophage migration inhibitory factor is produced by many organs and tissues during embryonic and fetal development and promotes lung development in mice (25–27). In humans, MIF is present in the amniotic fluid and is expressed in the fetal membranes of the placenta and in the fetal and neonatal lungs (28–31). Interestingly, circulating levels of MIF are at least 10-fold higher in healthy term newborns than in adults (30, 32, 33). We recently reported that high expression levels of MIF sustain innate immune responses of newborn monocytes and counter-regulate the activity of adenosine and prostaglandin E2, two immunosuppressive mediators produced at high levels by the placenta (33). Thus, MIF may play an important role in the developmental regulation of immune responses (33).

d-dopachrome tautomerase (DDT, also known as MIF-2) is the second member of the MIF protein superfamily (34). DDT and MIF are encoded by adjacent genes and share 34% amino acid identity in humans. DDT is constitutively expressed in the lungs, kidney, liver, spleen, heart, intestine, brain, and immune cells and circulates in the low nanograms per milliliter range in healthy individuals (35). Like MIF, DDT forms homotrimers and binds to the MIF receptor CD74. MIF and DDT induce similar signaling mechanisms and have overlapping biological activities, although much less is known about DDT than MIF. Blood levels of DDT are elevated in burn, septic, and cancer patients, and during cardiac surgery, and correlate with disease severity and clinical outcome (35–37). DDT, but not MIF, is a valuable biomarker to predict clinical outcome and sepsis occurrence after burn injury (36). So far, circulating levels of DDT in newborns and children have not been reported.

We hypothesized that levels of both MIF and DDT would be elevated in early life, at a period critical for the development of the lungs and the immune system, and that MIF and DDT could be attractive biomarker candidates in newborns. Concentrations of MIF and DDT were determined in a large cohort of different age groups including fetuses, preterm and term newborns, infants, children, and adults to get more insights into the developmental profile of MIF and DDT and establish reference intervals. Moreover, we correlated MIF and DDT levels with inflammatory and clinical parameters and outcome in very preterm infants who are at risk of developing severe complications including bronchopulmonary dysplasia (BPD) and late-onset neonatal sepsis (LOS).

## Subjects and Methods

### Subjects and Source of Plasma Samples

Plasma samples were obtained from 307 subjects from 5 groups. The first group comprised 15 fetuses in which blood samples were collected by cordocentesis prior to therapeutic abortions performed in the context of congenital malformations or chromosomal abnormalities (38). This study was approved by the Ethics Committee of the Erasme Hospital (Brussels, Belgium). The second group comprised 60 healthy term and 34 preterm neonates born at the University Hospital of Lausanne, Switzerland. Infants with congenital malformations, chromosomal abnormalities, perinatal asphyxia, and maternal, fetal, or neonatal infection were excluded. Cord blood was collected from the umbilical artery (UA) and the umbilical vein (UV) after delivery of the placenta. Peripheral blood was collected on postnatal day 4 in 12 healthy term newborns. The third group comprised 17 infants (aged between 1 and 12 months) and 73 children (aged between 1 and 16 years) admitted for elective surgery at the Department of Pediatrics, University Hospital of Lausanne, Switzerland (33, 39). Infants and children with acute or chronic infection, severe underlying disease, or chromosomal abnormality were excluded from the study. Peripheral blood was collected by venous puncture prior to surgery. The surgical procedures are listed in Table S1 in Supplementary Material. The fourth group included 58 healthy adult volunteers (>16 years old). In groups 1–4, heparinized blood samples were collected, and plasma was stored at −80°C until analysis. A complete blood count was performed in UV blood from 25 patients from group 2, using a Beckman Coulter ACT diff analyzer. The study with second, third, and fourth groups was approved by the Cantonal Ethics Committee of Vaud (Lausanne, Switzerland). The fifth group comprised 50 premature infants born before 32 weeks of gestation at the University Hospital of Zurich, Switzerland (40). Infants with complex congenital malformations, chromosomal abnormalities, and those who died within the first week after admission were excluded from the study. BPD was defined as requirement for supplemental oxygen for the first 28 days of life. LOS was defined as signs or symptoms of infection occurring at a postnatal age over 72 h in an infant with positive blood cultures and treated for at least 5 days with antibiotics. Blood samples were collected in EDTA tubes on postnatal day 6, and plasma was stored at −20°C until analysis. The study was approved by the Cantonal Ethics Committee of Zurich. All subjects from the five groups or their legal guardians gave written informed consent in accordance with the Declaration of Helsinki.

### Measurement of MIF Levels in Plasma

Macrophage migration inhibitory factor levels were measured by enzyme-linked immunosorbent assay (ELISA), using the four-span approach, as previously described (41). Briefly, 96-well microtiter plates were coated with a duck anti-chicken antibody. Anti-human MIF polyclonal antibodies raised in chicken and rabbit were used as capture and trapping antibodies. A horseradish peroxidase-labeled goat anti-rabbit antibody was used for detection. Recombinant human MIF was used as a standard. The analytic sensitivity of the human MIF ELISA was 39 pg/ml. Intrarun and interrun coefficients of variation were 6 and 12%, respectively.

### Measurement of MIF Secretion by Leukocyte Subsets

Umbilical cord blood mononuclear cells from healthy term newborns and peripheral blood mononuclear cells from adult volunteers were obtained as previously described (33, 42). Neutrophils, monocytes, B cells, and T cells were isolated from mononuclear cells using magnetic microbeads (Miltenyi Biotec) coupled to antibodies directed against CD15, CD14, CD19, and CD3, respectively. Cells (10^5^/well in 96-well plates) were cultured in RPMI medium 1640 supplemented with 10% fetal calf serum. MIF levels were measured by ELISA (R&D Systems) in cell culture supernatant collected at 24 and 48 h.

### Measurement of DDT Levels in Plasma

d-dopachrome tautomerase levels were measured by ELISA as previously described (35). Briefly, 96-well microtiter plates were coated with rabbit polyclonal antibodies raised against recombinant human DDT. Wells were washed and blocked in 1% BSA and 1% sucrose. Samples were added and incubated for 2 h, followed by biotinylated anti-DDT antibody and a streptavidin–HRP conjugate. DDT concentrations were calculated by extrapolation from a sigmoidal quadratic standard curve obtained using recombinant human DDT used as a standard. The analytic sensitivity of the DDT ELISA was 15 pg/ml. Intrarun and interrun coefficients of variation were 8 and 10%, respectively.

### Measurement of TNF, IL-1β, IL-6, IL-8, and Vascular Endothelial Growth Factor (VEGF) Levels in Plasma

Levels of TNF, IL-1β, IL-6, IL-8, and VEGF were measured using a ProcartaPlex panel (Affimetrix eBioscience) and a Luminex 200 System analyzer (Luminex Corporation). Concentrations were calculated by extrapolation from a sigmoidal quadratic standard curve obtained using recombinant human standards. The limits of quantification of TNF, IL-1β, IL-6, IL-8, and VEGF were 6.52, 2.05, 8.15, 2.17, and 5.74 pg/ml, respectively.

### Statistical Analyses

Data are presented as median and interquartile range (IQR) and as mean and SD. Comparisons between groups were performed by Kruskal–Wallis and Mann–Whitney tests. Correlations between MIF and DDT levels and clinical variables were assessed by the non-parametric Spearman test. Exploratory multivariate analyses were performed on the composite outcome of BPD and/or LOS using logistic regression. Covariates associated with the outcome at a *P* value level of <0.1 were considered. Final models were adjusted for birthweight, given the strong influence of birthweight on outcomes in premature infants (43). Findings were considered statistically significant when *P* < 0.05. Statistical analyses were performed using Prism 7 (GraphPad Software, La Jolla, CA, USA) and IBM SPSS Statistics 22.

## Results

### MIF and DDT Plasma Levels in Different Age Groups

**Figure 1:**
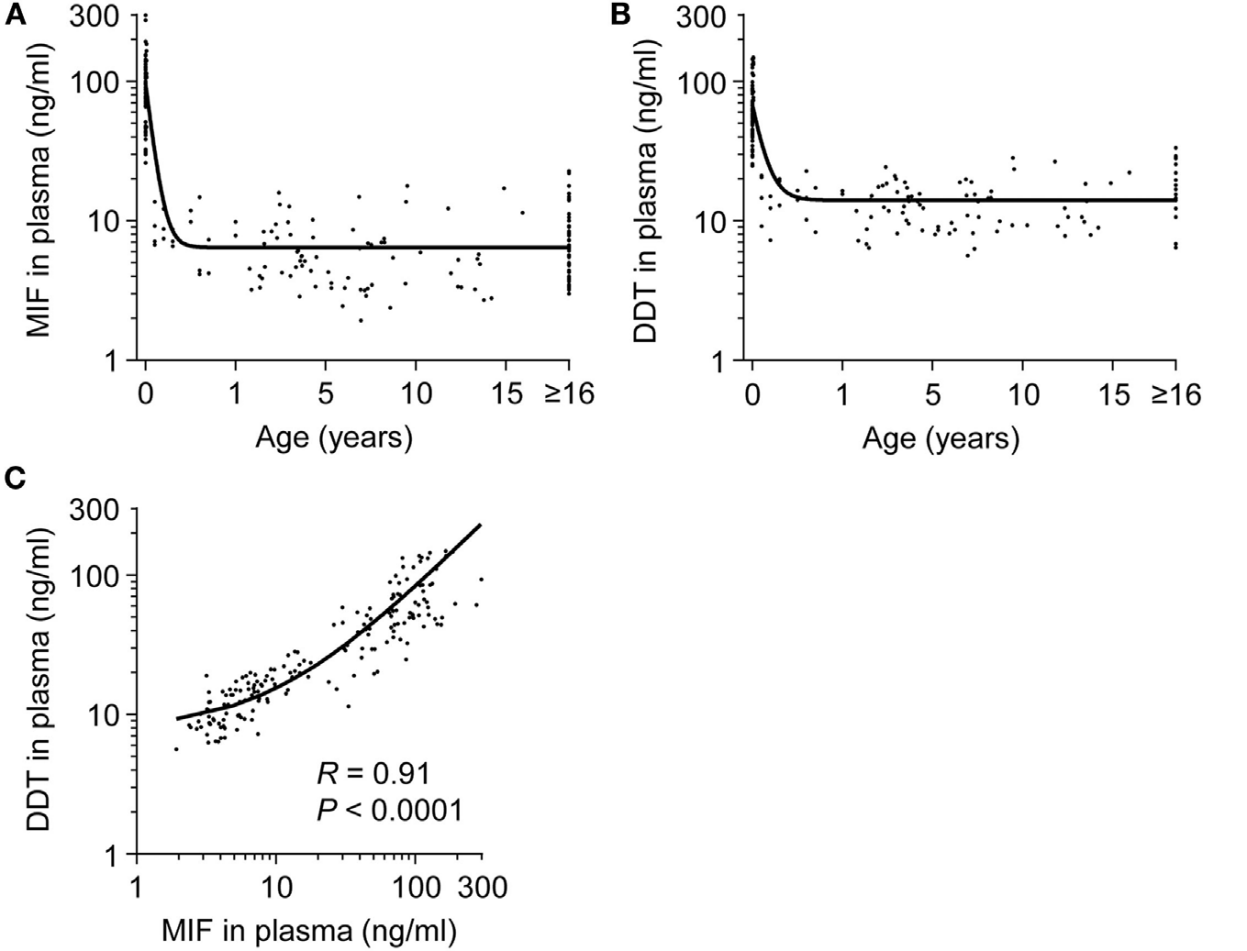
**Plasma concentrations of macrophage migration inhibitory factor (MIF) and d-dopachrome tautomerase (DDT) in healthy subjects from birth to adulthood**. MIF **(A)** and DDT **(B)** plasma concentrations in the umbilical vein of 60 healthy term newborns and in the peripheral blood of 12 healthy term newborns, 17 infants, 73 children, and 58 adults (19 adults for DDT). The regression lines are shown. **(C)** Scatterplot comparing MIF to DDT concentrations in paired measurements from all age groups. The regression line is shown. Data from **(A)** was obtained from Ref. (33).

Plasma concentrations of MIF and DDT were measured in 15 fetuses (at 20–36 weeks gestation), 60 term newborns (at birth), 12 term newborns (on postnatal day 4), 17 infants (1–12 months old), 73 children (1–16 years old), and 58 adults (>16 years old). Strikingly, MIF concentrations were 5- to 20-fold higher in fetuses (median 48.9 ng/ml, IQR 33.2–65.0) and in healthy term newborns at birth (82.6 ng/ml, 66.1–115.4) and on postnatal day 4 (109.5 ng/ml, 76.5–159.5) than in infants (7.4 ng/ml, 6.6–10.8), children (5.2 ng/ml, 3.4–7.7), and adults (5.7 ng/ml, 4.0–8.3) (*P* < 0.05; Table 1; Figure 1A). Similarly, DDT concentrations were 3- to 10-fold higher in healthy term newborns at birth (52.0 ng/ml, 43.9–72.4) and on postnatal day 4 (121.6 ng/ml, 74.1–137.3) than in infants (14.6 ng/ml, 11.2–19.6), children (12.5 ng/ml, 9.1–16.3), and adults (16.8 ng/ml, 14.1–25.4) (*P* < 0.05; Figure 1B). DDT concentrations in healthy term newborns on postnatal day 4 were also significantly higher than in fetuses (29.6 ng/ml, 18.9–45.6) (*P* < 0.05; Table 1). Overall, MIF and DDT concentrations were high in fetuses, even higher in term newborns at birth and on postnatal day 4, and decreased to the adult levels during the first months of life. A regression analysis of paired measurements revealed a strong correlation between MIF and DDT concentrations (*R* = 0.91, *P* < 0.0001; Figure 1C).

**Table 1:** **Macrophage migration inhibitory factor (MIF) and d-dopachrome tautomerase (DDT) plasma concentrations in different age groups**.

	*N*	MIF (ng/ml)		DDT (ng/ml)	
		Median [interquartile range (IQR)]	Mean (SD)	Median (IQR)	Mean (SD)
Fetuses^a^	15	48.9^c^ (33.2–65.0)	51.9 (23.6)	29.6 (18.9–45.6)	31.5 (14.5)
Term newborns (day 0)^b^	60	82.6^c^ (66.1–115.4)	91.9 (51.2)	52.0^c^ (43.9–72.4)	61.1 (27.1)
Term newborns (day 4)	12	109.5^c^ (76.5–159.5)	114.1 (43.1)	121.6^c^^,^^d^ (74.1–137.3)	107.8 (36.0)
Infants (1–12 months)	17	7.4 (6.6–10.8)	8.5 (3.2)	14.6 (11.2–19.6)	15.0 (4.7)
Children (1–16 years)	73	5.2 (3.4–7.7)	6.3 (3.7)	12.5 (9.1–16.3)	13.3 (5.1)
Adults (>16 years)	58^e^	5.7 (4.0–8.3)	7.1 (4.5)	16.8 (14.1–25.4)	18.3 (7.6)

*^a^Median gestational age: 27 weeks (IQR: 25–29)*.

*^b^Blood was collected from the umbilical vein*.

*^c^P < 0.05 versus infants, children, and adults*.

*^d^P < 0.05 versus fetuses, infants, children, and adults*.

*^e^DDT levels were measured in 19 adults*.

### MIF and DDT Levels in Umbilical Cord Blood at Birth

**Figure 2:**
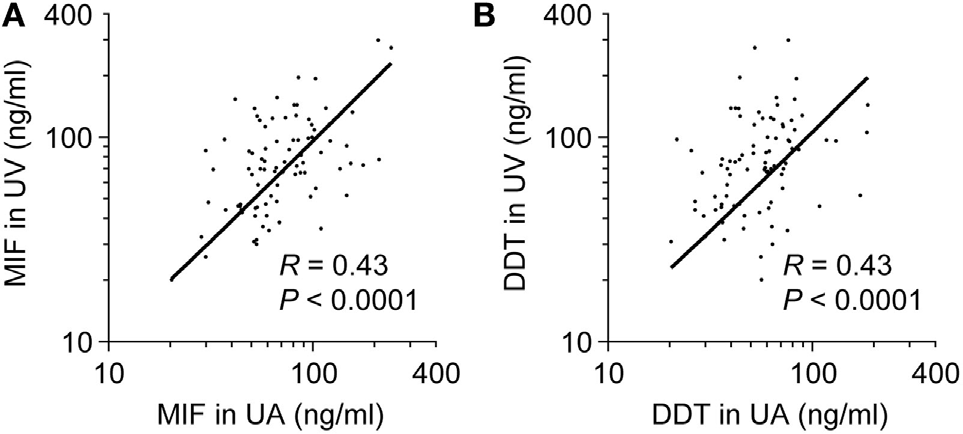
**Comparison of umbilical artery (UA) to umbilical vein (UV) macrophage migration inhibitory factor (MIF) and d-dopachrome tautomerase (DDT) plasma concentrations**. Scatterplot comparing MIF **(A)** and DDT **(B)** plasma concentrations in UA and UV in 86 **(A)** and 88 **(B)** paired measurements from preterm and term newborns. The regression lines are shown.

To determine whether high concentrations of MIF and DDT in newborns are of fetal or placental origin, we compared MIF and DDT concentrations in the UA that drives blood flowing from the fetus to the placenta with that in the UV that drives blood flowing from the placenta to the fetus (44). The clinical characteristics of the 34 preterm and 60 term newborns selected for these measures are presented in Table 2. Median (IQR) gestational age was 34 (32–36) and 39 (39–40) weeks for preterm and term newborns, respectively. MIF and DDT concentrations in the UA correlated with the concentrations measured in the UV (*R* = 0.43, *P* < 0.0001 for both MIF and DDT; Figure 2) and were not different from each other (*P* = 0.33 for MIF and *P* = 0.71 for DDT). Thus, high levels of MIF and DDT in the circulation of newborns can be of fetal or placental origin.

**Table 2:** **Clinical characteristics of the newborns included in measurements of macrophage migration inhibitory factor and d-dopachrome tautomerase concentrations in umbilical cord blood**.

	Preterm newborns (*n* = 34)	Term newborns (*n* = 60)
Maternal group B streptococcal status, *n* (%)		
Positive	3 (9)	6 (10)
Negative	25 (74)	39 (65)
Unknown	6 (18)	15 (25)
Intrapartum antibiotic prophylaxis, *n* (%)	7 (21)	6 (10)
Antenatal steroids, *n* (%)	31 (91)	3 (5)
Mode of delivery, *n* (%)		
Vaginal	6 (18)	30 (50)
Emergency cesarean section	16 (47)	3 (5)
Elective cesarean section	12 (35)	27 (45)
Male gender, *n* (%)	22 (65)	26 (43)
Median gestational age at birth, weeks [interquartile range (IQR)]	34 (32–36)	39 (39–40)
Median birthweight, g (IQR)	1,960 (1,665–2,295)	3,440 (3,280–3,670)
Median umbilical artery pH (IQR)	7.30 (7.26–7.33)	7.27 (7.21–7.30)
Median umbilical vein pH (IQR)	7.35 (7.31–7.37)	7.33 (7.29–7.37)
Median 1 min Apgar score (IQR)	9 (7–9)	9 (9–9)
Median 5 min Apgar score (IQR)	9 (8–9)	10 (9–10)
Median 10 min Apgar score (IQR)	10 (9–10)	10 (10–10)

**Figure 3:**
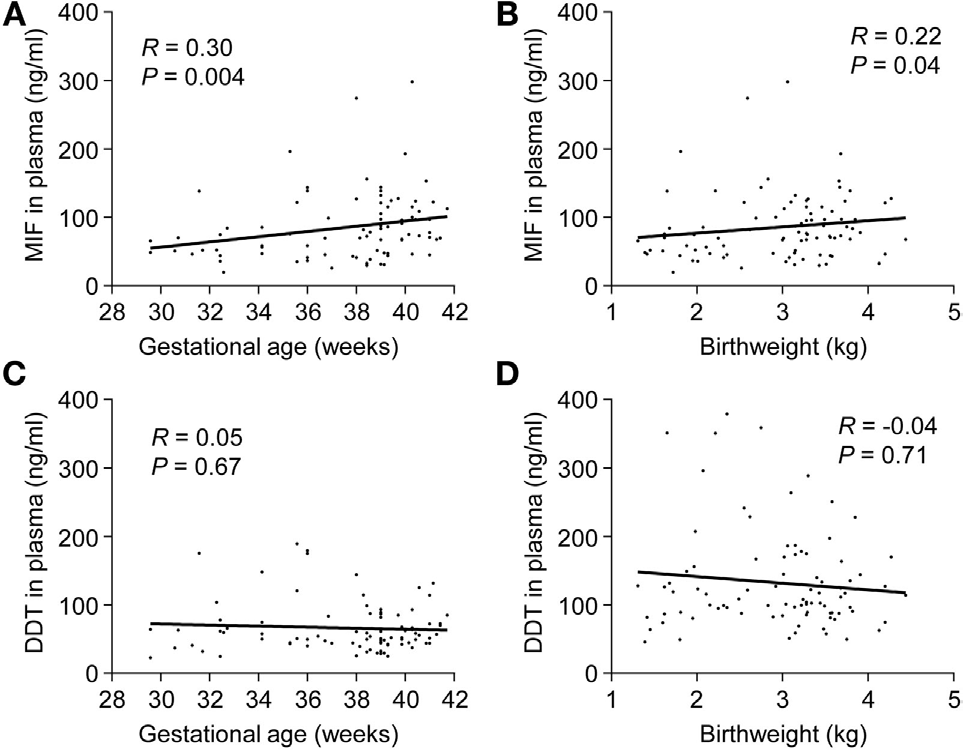
**Umbilical cord blood concentrations of macrophage migration inhibitory factor (MIF) and d-dopachrome tautomerase (DDT) in preterm and term newborns**. Scatterplot showing MIF **(A,B)** and DDT **(C,D)** concentrations in the umbilical vein versus gestational age and birthweight of 30 preterm and 60 term newborns. The regression lines are shown.

In preterm newborns, median UV MIF and DDT concentrations were 58.3 and 58.7 ng/ml, respectively (Table 3). MIF concentrations in UV correlated with gestational age (*R* = 0.30, *P* = 0.004) and birthweight (*R* = 0.22, *P* = 0.04), while no such correlation was found for DDT (Figure 3). MIF and DDT concentrations in UV were not influenced by gender, mode of delivery, maternal group B streptococcal status, intrapartum antibiotic prophylaxis, UA and UV pH, and Apgar scores at 1, 5, and 10 min.

**Table 3:** **MIF and DDT plasma concentrations in the UV and UA of preterm and term newborns**.

	MIF (ng/ml)			DDT (ng/ml)		
	*N*	Median (IQR)	Mean (SD)	*N*	Median (IQR)	Mean (SD)
Preterm newborns, UV	29	58.3 (45.6–84.9)	72.0 (40.4)	30	58.7 (44.3–88.6)	76.2 (49.2)
Preterm newborns, UA	31	61.7 (51.8–95.1)	72.8 (32.5)	32	55.6 (39.7–74.9)	65.0 (37.6)
Term newborns, UV	60	82.6 (66.1–115.4)	91.9 (51.2)	60	52.0 (43.9–72.4)	61.1 (27.1)
Term newborns, UA	59	66.9 (53.0–95.7)	80.7 (44.8)	60	58.9 (41.5–71.8)	60.1 (27.0)

*UV, umbilical vein; UA, umbilical artery; IQR, interquartile range; MIF, macrophage migration inhibitory factor; DDT, d-dopachrome tautomerase*.

### Associations between MIF and DDT Levels and Blood Cells

**Figure 4:**
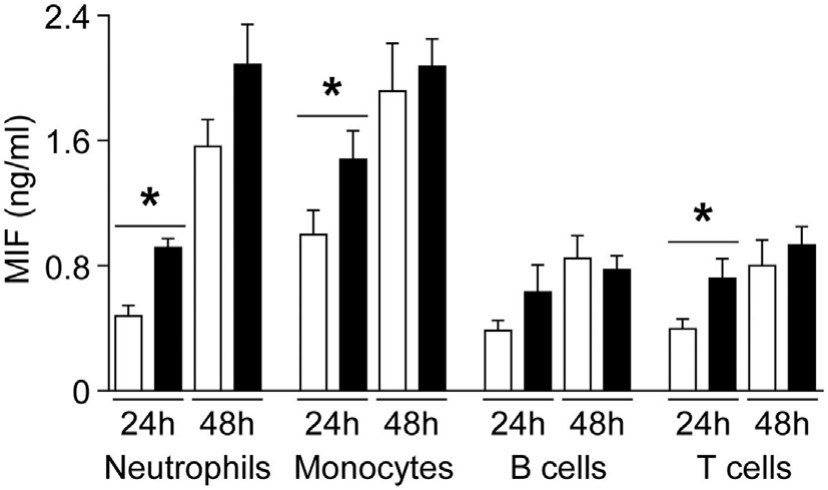
**Macrophage migration inhibitory factor (MIF) secretion by newborn and adult leukocyte subsets**. Neutrophils, monocytes, B cells, and T cells from healthy term newborns (white bars) and adult volunteers (black bars) were cultured in RPMI medium supplemented with 10% FCS. MIF levels were measured by enzyme-linked immunosorbent assay in supernatant collected at 24 and 48 h. Data represent means ± SEM of five to nine independent experiments performed in triplicates (*P < 0.05).

To evaluate whether MIF and DDT levels are associated with specific cell types, we performed a complete blood count in UV blood of 25 healthy term newborns. UV MIF levels were not influenced by total leukocyte count, lymphocytes, monocytes, granulocytes, platelets, erythrocytes, hemoglobin, and hematocrit. UV DDT levels negatively correlated with monocyte levels (*R* = −0.46, *P* = 0.02), but no correlation was observed with other cell types. Next, we investigated whether leukocyte subsets isolated from newborns and adults release different amounts of MIF. After 24 h of culture, neutrophils, monocytes, and T cells from adults released higher amounts of MIF compared to newborn cells (Figure 4). However, no difference in MIF secretion between adult and newborn neutrophils, monocytes, B cells, and T cells was observed at 48 h. Therefore, the large interindividual variations in MIF and DDT observed at birth are not associated with differences in the main demographic and clinical characteristics or with the predominance of specific blood cell subtype. Moreover, newborn leukocyte subsets do not release higher levels of MIF *in vitro* compared to adult leukocyte subsets.

### Associations between MIF and DDT Levels, VEGF, and Cytokines

**Figure 5:**
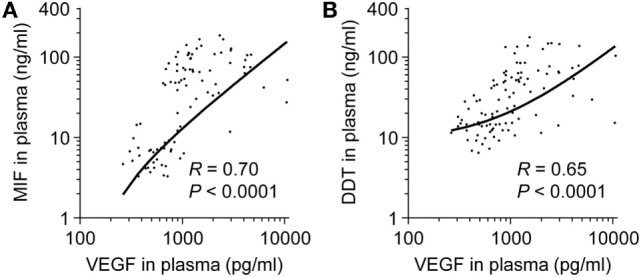
**Comparison of macrophage migration inhibitory factor (MIF) and d-dopachrome tautomerase (DDT) to vascular endothelial growth factor (VEGF) plasma concentrations**. Scatterplot comparing MIF **(A)** and DDT **(B)** to VEGF plasma concentrations in paired measurements from 14 fetuses, 14 preterm and 18 term newborns (umbilical vein), and from peripheral blood of 11 term newborns (on postnatal day 4), 17 infants, 14 children, and 15 adults. The regression lines are shown.

Macrophage migration inhibitory factor and DDT are induced during hypoxia and inflammation (35, 45). We tested whether MIF and DDT levels correlate with VEGF, an angiogenic protein upregulated by hypoxia-inducible factor (HIF)-1α under hypoxic conditions, and inflammatory cytokines during development. Circulating levels of VEGF were higher in fetuses (median 1,423 pg/ml, IQR 1,065–4,102) and in preterm newborns at birth (1,551 pg/ml, 1,064–2,441) compared to infants (676 pg/ml, 527–810), children (485 pg/ml, 360–639), and adults (486 pg/ml, 420–665) (*P* < 0.05). In term newborns, VEGF levels were higher at birth (1,090 pg/ml, 825–1,918) and on postnatal day 4 (1,476 pg/ml, 1,161–2,512) compared to children (*P* = 0.05 and *P* < 0.05 versus VEGF levels at birth and on postnatal day 4) and adults (*P* = 0.06 and *P* < 0.05 versus VEGF levels at birth and on postnatal day 4). Overall, VEGF strongly correlated with MIF and DDT (*R* = 0.70, *P* < 0.0001 for MIF and *R* = 0.65, *P* < 0.0001 for DDT; Figure 5). Median concentrations of IL-1β, IL-6, and TNF were below the limit of quantification in all age groups confirming that, contrary to MIF and DDT, circulating levels of classical inflammatory cytokines are very low in the absence of infectious or inflammatory disease. Median levels of the IL-8 were 3.9 pg/ml (1.1–15.5) in fetuses and 6.1 pg/ml (1.1–49.2) in the UV of preterm newborns but were below the detection limit in term newborns, infants, children, and adults.

### MIF and DDT Levels in Peripheral Blood of Very Preterm Infants

**Figure 6:**
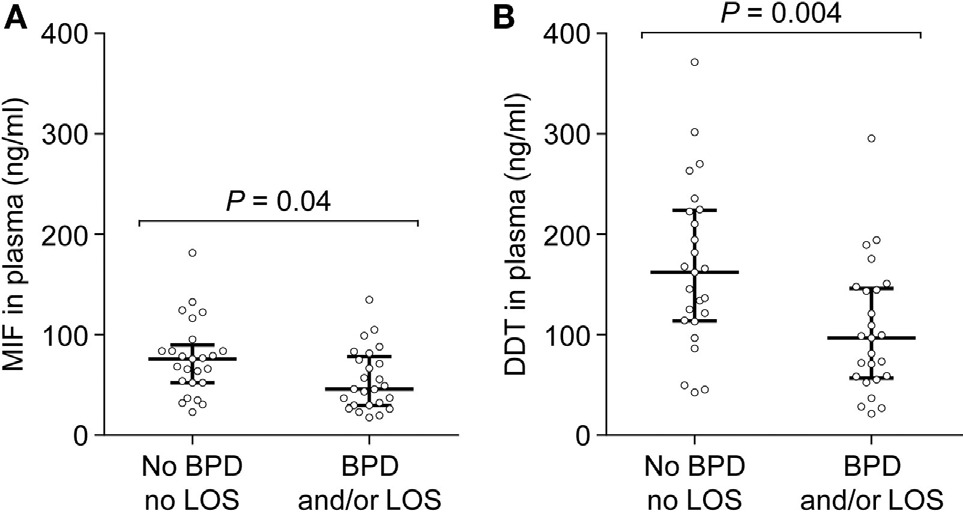
**Macrophage migration inhibitory factor (MIF) and d-dopachrome tautomerase (DDT) plasma concentrations in peripheral blood of very preterm newborns**. MIF **(A)** and DDT **(B)** plasma concentrations were measured on postnatal day 6 in 25 very preterm newborns who subsequently developed bronchopulmonary dysplasia and/or late-onset sepsis and in 25 preterm newborns who did not develop these complications. The median and interquartile range are shown.

Preterm newborns are at risk of developing severe complications such as BPD and LOS. Association between MIF and DDT expression levels and outcome of preterm birth was explored by measuring MIF and DDT concentrations in the plasma of 50 very preterm infants (gestational age 26–31 weeks) on postnatal day 6, prior to the development of BPD and LOS. Clinical characteristics of the patients are presented in Table 4. Among the 50 patients, 25 (50%) developed BPD and/or LOS, with 21 (42%) developing BPD, and 14 (28%) developing LOS. One patient died at 14 days of age, following LOS. MIF and DDT concentrations (median, IQR) were lower in infants who developed BPD and/or LOS than in those who did not develop these complications (MIF: 45.9 ng/ml, 29.8–78.2 versus 75.7 ng/ml, 52.1–89.6, *P* = 0.04; DDT: 96.8 ng/ml, 56.7–146.2 versus 162.1 ng/ml, 113.6–223.6, *P* = 0.004; Figures 6A,B). Exploratory multivariate analyses adjusted for birthweight confirmed that lower concentrations of MIF and DDT were associated with an increased risk of the composite outcome of BPD and/or LOS in this cohort (*P* = 0.054 and *P* = 0.017, respectively).

**Table 4:** **Clinical characteristics of 50 preterm newborns included in measurements of macrophage migration inhibitory factor and d-dopachrome tautomerase concentrations in peripheral blood on postnatal day 6**.

	Preterm newborns without bronchopulmonary dysplasia (BPD) and/or late-onset sepsis (*n* = 25)	Preterm newborns with BPD and/or late-onset sepsis (*n* = 25)	*P* value
Antenatal steroids, *n* (%)	24 (96)	22 (88)	0.61
Prelabor rupture of membranes, *n* (%)	7 (28)	7 (28)	1.00
Preclampsia, *n* (%)	6 (24)	2 (8)	0.29
Histological chorioamnionitis, *n* (%)	12 (48)	10 (40)	0.77
Vaginal delivery, *n* (%)	3 (12)	3 (12)	1.00
Male gender, *n* (%)	11 (44)	12 (48)	1.00
Median gestational age at birth, weeks [interquartile range (IQR)]	31 (30–31)	27 (26–27)	<0.0001
Median birthweight, g (IQR)	1,510 (1,270–1,755)	870 (795–1,065)	<0.0001
Median umbilical artery pH (IQR)	7.35 (7.32–7.37)	7.34 (7.30–7.40)	0.99
Median 5 min Apgar score (IQR)	7 (6–9)	5 (3–7)	0.004
Median 10 min Apgar score (IQR)	8 (8–9)	7 (6–8)	0.13
Mechanical ventilation, *n* (%)	6 (24)	20 (80)	0.001
BPD^a^, *n* (%)	0	21 (84)	<0.0001
Medically treated patent ductus arteriosus, *n* (%)	6 (24)	18 (72)	0.002
Surgically treated patent ductus arteriosus, *n* (%)	0	8 (32)	0.008
Blood culture proven early-onset sepsis, *n* (%)	0	1 (4)	1.00
Blood culture proven late-onset sepsis, *n* (%)	0	14 (56)	0.0001
Necrotizing enterocolitis^b^, *n* (%)	0	2 (8)	0.50
Retinopathy of prematurity, *n* (%)	0	6 (24)	0.03
Intraventricular hemorrhage, *n* (%)	1 (4)	12 (48)	0.06
Periventricular leukomalacia, *n* (%)	0	3 (12)	NA
Death, *n* (%)	0	1 (4)	NA

*^a^Defined as requirement for supplemental oxygen for more than 28 days*.

*^b^Bell stage ≥2*.

*NA, not applicable*.

**Figure 7:**
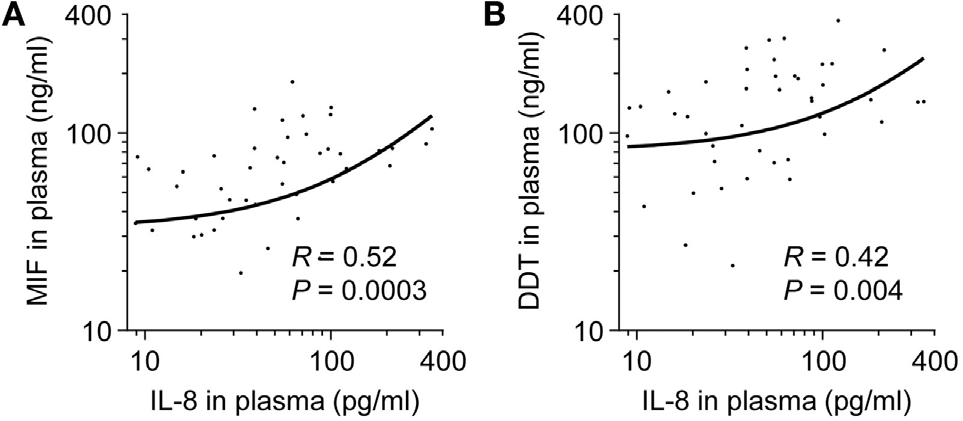
**Comparison of macrophage migration inhibitory factor (MIF) and d-dopachrome tautomerase (DDT) to IL-8 plasma concentrations in very preterm newborns**. Scatterplot comparing MIF **(A)** and DDT **(B)** to IL-8 plasma concentrations in paired measurements from 50 very preterm newborns. The regression lines are shown.

Levels of VEGF, IL-1β, IL-6, IL-8, and TNF were measured in the 50 very preterm newborns and were compared to MIF and DDT levels. VEGF concentrations were 2,582 pg/ml (826–5,293) and did not correlate with MIF or DDT concentrations (*R* = 0.21, *P* = 0.14 for MIF and *R* = 0.25, *P* = 0.1 for DDT). Median concentrations of IL-1β, IL-6, and TNF were below the limit of quantification. IL-8 concentrations were 42.6 pg/ml (19.8–89.8) and correlated with MIF and DDT concentrations (*R* = 0.52, *P* = 0.0003 for MIF and *R* = 0.42, *P* = 0.004 for DDT; Figures 7A,B).

## Discussion

This is the first study investigating MIF and DDT plasma levels in a large cohort of individuals ranging from fetuses to adults. Both mediators circulate at baseline in healthy subjects, with a strong correlation between MIF and DDT plasma concentrations and striking age-dependent fluctuations. Highest concentrations of >100 ng/ml were measured on postnatal day 4, a situation unique for cytokines that usually do not reach such levels except in extreme pathological situations. MIF and DDT levels dropped 7- to 20-fold to reach adult levels during the first months of life.

Plasma levels of MIF and DDT were high in fetuses and increased further at birth, suggesting that both the fetal environment and the adaptive changes occurring during delivery could contribute to MIF and DDT circulating pools in newborns. Whether MIF and DDT mostly originate from the fetus or the placenta remains an open question given that their concentrations in the blood flowing in the UA and UV were comparable. The fetal environment is characterized by low oxygen tension and high concentrations of steroid hormones such as estradiol and progesterone that could both stimulate MIF and DDT production by the placenta and the fetus. Indeed, *MIF* and *DDT* are hypoxia-inducible genes (45, 46). VEGF promotes fetal vascular and pulmonary development and is tightly regulated by HIF activity (47). VEGF levels were higher in fetuses and newborns, compared to children and adults, and strongly correlated with MIF and DDT levels. This is in line with previous studies showing that MIF induces secretion of VEGF in several cancer cell lines (48–50) and that MIF-deficient mice express reduced levels of VEGF in the lungs (27). Elevated MIF concentrations at birth were not associated with a predominance of specific leukocyte subsets or an increased capacity of newborn leukocytes to secrete MIF *in vitro*. However, steroids produced by the placenta and the fetal adrenal gland can stimulate MIF secretion by newborn monocytes (33). Moreover, circulating leukocyte counts are at least twofold higher at birth than in adults (51). Therefore, a combination of high circulating levels of steroid hormones acting on a high number of leukocytes and a relatively low oxygen tension *in utero* could contribute to the strikingly elevated levels of MIF and DDT observed at birth.

The immune system is shaped by particular constrains during the fetal and neonatal periods, as illustrated by age-dependent variations in blood concentrations of pro-inflammatory and anti-inflammatory mediators (52). We did not detect measurable levels of IL-1β, IL-6, IL-8, and TNF in healthy term newborns, suggesting that elevated MIF and DDT levels at birth do not reflect systemic inflammation. In contrast, estradiol, progesterone, adenosine, and prostaglandins circulate at high concentrations perinatally (42, 53–55) and provide a skewed anti-inflammatory milieu that preserves gestation and contributes to maintain tolerance to postnatal microbial colonization. Yet, this comes at the expense of weakening the ability to mount efficient immune responses against pathogens during the neonatal period (52, 56). Indeed, newborns are particularly susceptible to development of severe bacterial infections (57, 58). We have recently shown that MIF sustains microbial product-induced cell activation and cytokine production and counterregulates adenosine and prostaglandin E_2_-mediated immune suppression in newborn monocytes (33). Therefore, high levels of MIF, and possibly DDT, may be part of a counterregulatory mechanism to balance innate immune responses perinatally.

Macrophage migration inhibitory factor promotes lung development and protects preterm mice from neonatal respiratory distress syndrome and hyperoxia-induced lung injury (27, 59). We report lower expression of MIF and DDT on postnatal day 6 in the circulation of very preterm newborns who subsequently developed BPD and/or LOS, two major complications of preterm birth. This is in line with the observation that MIF levels in tracheal aspirates obtained within the first 2 days of life are reduced in preterm infants who develop BPD (27, 30). Studies on the role of DDT have been limited to adults in humans and animals so far (34–36, 45, 60–62), and we do not known to which extend DDT impacts, like MIF, on lung development and innate immune responses in early life. Interestingly, IL-8 was detected in fetuses and preterm newborns, but not in term infants, children, and adults. In very preterm newborns, IL-8 levels measured on postnatal day 6 correlated with MIF and DDT. The frequency of circulating IL-8 producing CD4 T cells that can activate protective immune responses is fivefold higher in neonates than in adults (63). While MIF can stimulate IL-8 production by a variety of cell types including T cells (49, 50, 64), it remains to be determined whether MIF is implicated in T cell function in newborns. Overall, our results support a role for MIF in host defenses during the neonatal period.

Interindividual variations of circulating MIF levels have been analyzed in the context of *MIF* polymorphisms in patients with inflammatory, autoimmune, and infectious diseases (14, 15). Yet, no polymorphisms of the *DDT* gene have been reported. While carriage of high expression *MIF* alleles was generally associated with more severe complications and poor outcome, it has also been associated with survival in patients with community-acquired pneumonia (17, 65, 66). Our findings of higher MIF levels in infants who did not develop BPD or LOS are consistent with a previous study showing an association between carriage of a high expression *MIF* allele and a lower incidence of BPD in preterm newborns (30). We did not determine the *MIF* genotype of the very preterm newborns because genetic material was unavailable and because such a study would be strongly underpowered considering *MIF* allele frequencies and patient number (14, 16, 17). A nationwide pediatric sepsis cohort study has been launched in Switzerland and will address the relationship between MIF and DDT gene polymorphisms and expression levels and clinical outcome (58, 67).

The strengths of this study include its large size and the fact that it represents the first evaluation of MIF and DDT concentrations throughout life. Few studies, and none in the pediatric population, have investigated DDT plasma concentrations and have compared MIF to DDT levels (35–37). This study has several limitations. For ethical reasons, it was not possible to obtain sequential blood samples over a prolonged period in newborns, infants, and children. To establish age-specific reference values, we pooled samples from several cohorts covering a wide age range. Fetal samples were obtained prior to therapeutic abortions for a range of congenital malformations or chromosomal abnormalities, minimizing the possible impact of a specific condition on MIF and DDT levels (38). Samples were obtained in infants and children undergoing general anesthesia for elective surgery. As blood collection occurred upon induction of anesthesia, it is unlikely that physiological changes due to surgery could have altered MIF and DDT levels.

In conclusion, circulating concentrations of MIF and DDT are tightly correlated and change significantly with age, with the highest levels in newborns and the lowest levels in adults. The present study provides age-specific plasma concentrations of MIF and DDT in healthy individuals, which may serve to establish cutoff values in future studies. Moreover, our observations open the venue for future studies investigating whether MIF and DDT levels during the first week of life could be used as biomarkers to help predicting the occurrence of later complications in preterm newborns. Future studies should also examine associations between functional *MIF* polymorphisms and susceptibility to neonatal and pediatric sepsis and BPD, and severity of these diseases.

## Author Contributions

TR and EG designed the study, interpreted the data, and drafted and revised the manuscript. LS and TC analyzed and interpreted the data and revised the manuscript. MW, AS, SW, DV, and PM acquired and analyzed the data and revised the manuscript. FS, LL, and RB provided new reagents and analytic tools and revised the manuscript.

## Conflict of Interest Statement

The authors declare that there are no competing interests regarding the publication of this paper. The reviewer YM and handling Editor declared their shared affiliation, and the handling Editor states that the process nevertheless met the standards of a fair and objective review.

## References

[1] Calandra T Roger T. Macrophage migration inhibitory factor: a regulator of innate immunity. Nat Rev Immunol (2003) 3(10):791–800. 10.1038/nri1200 14502271 PMC7097468

[2] Bucala R Donnelly SC. Macrophage migration inhibitory factor: a probable link between inflammation and cancer. Immunity (2007) 26(3):281–5. 10.1016/j.immuni.2007.03.005 17376392

[3] Leech M Metz C Hall P Hutchinson P Gianis K Smith M Macrophage migration inhibitory factor in rheumatoid arthritis: evidence of proinflammatory function and regulation by glucocorticoids. Arthritis Rheum (1999) 42(8):1601–8. 10.1002/1529-0131(199908)42:8<1601::AID-ANR6>3.0.CO;2-B 10446857

[4] Foote A Briganti EM Kipen Y Santos L Leech M Morand EF. Macrophage migration inhibitory factor in systemic lupus erythematosus. J Rheumatol (2004) 31(2):268–73. 14760795

[5] Shimizu T Nishihira J Mizue Y Nakamura H Abe R Watanabe H High macrophage migration inhibitory factor (MIF) serum levels associated with extended psoriasis. J Invest Dermatol (2001) 116(6):989–90. 10.1046/j.0022-202x.2001.01366.x 11407993

[6] Shimizu T Abe R Ohkawara A Mizue Y Nishihira J. Macrophage migration inhibitory factor is an essential immunoregulatory cytokine in atopic dermatitis. Biochem Biophys Res Commun (1997) 240(1):173–8. 10.1006/bbrc.1997.7633 9367905

[7] Murakami H Akbar SM Matsui H Onji M. Macrophage migration inhibitory factor in the sera and at the colonic mucosa in patients with ulcerative colitis: clinical implications and pathogenic significance. Eur J Clin Invest (2001) 31(4):337–43. 10.1046/j.1365-2362.2001.00796.x 11298781

[8] Yamaguchi E Nishihira J Shimizu T Takahashi T Kitashiro N Hizawa N Macrophage migration inhibitory factor (MIF) in bronchial asthma. Clin Exp Allergy (2000) 30(9):1244–9. 10.1046/j.1365-2222.2000.00888.x 10971470

[9] Meyer-Siegler KL Bellino MA Tannenbaum M. Macrophage migration inhibitory factor evaluation compared with prostate specific antigen as a biomarker in patients with prostate carcinoma. Cancer (2002) 94(5):1449–56. 10.1002/cncr.10354 11920501

[10] Calandra T Echtenacher B Roy DL Pugin J Metz CN Hultner L Protection from septic shock by neutralization of macrophage migration inhibitory factor. Nat Med (2000) 6(2):164–70. 10.1038/72262 10655104

[11] Lehmann LE Novender U Schroeder S Pietsch T von Spiegel T Putensen C Plasma levels of macrophage migration inhibitory factor are elevated in patients with severe sepsis. Intensive Care Med (2001) 27(8):1412–5. 10.1007/s001340101022 11511957

[12] Bozza FA Gomes RN Japiassu AM Soares M Castro-Faria-Neto HC Bozza PT Macrophage migration inhibitory factor levels correlate with fatal outcome in sepsis. Shock (2004) 22(4):309–13. 10.1097/01.shk.0000140305.01641.c8 15377884

[13] Emonts M Sweep FC Grebenchtchikov N Geurts-Moespot A Knaup M Chanson AL Association between high levels of blood macrophage migration inhibitory factor, inappropriate adrenal response, and early death in patients with severe sepsis. Clin Infect Dis (2007) 44(10):1321–8. 10.1086/514344 17443469

[14] Renner P Roger T Calandra T. Macrophage migration inhibitory factor: gene polymorphisms and susceptibility to inflammatory diseases. Clin Infect Dis (2005) 41(Suppl 7):S513–9. 10.1086/432009 16237655

[15] Bucala R. MIF, MIF alleles, and prospects for therapeutic intervention in autoimmunity. J Clin Immunol (2013) 33(Suppl 1):S72–8. 10.1007/s10875-012-9781-1 22968741 PMC3548018

[16] Renner P Roger T Bochud PY Sprong T Sweep FC Bochud M A functional microsatellite of the macrophage migration inhibitory factor gene associated with meningococcal disease. FASEB J (2012) 26(2):907–16. 10.1096/fj.11-195065 21990375

[17] Savva A Brouwer MC Roger T Valls Seron M Le Roy D Ferwerda B Functional polymorphisms of macrophage migration inhibitory factor as predictors of morbidity and mortality of pneumococcal meningitis. Proc Natl Acad Sci U S A (2016) 113(13):3597–602. 10.1073/pnas.1520727113 26976591 PMC4822597

[18] Grieb G Merk M Bernhagen J Bucala R. Macrophage migration inhibitory factor (MIF): a promising biomarker. Drug News Perspect (2010) 23(4):257–64. 10.1358/dnp.2010.23.4.1453629 20520854 PMC3131110

[19] Morand EF Leech M Bernhagen J MIF: a new cytokine link between rheumatoid arthritis and atherosclerosis. Nat Rev Drug Discov (2006) 5(5):399–410. 10.1038/nrd2029 16628200

[20] Greven D Leng L Bucala R. Autoimmune diseases: MIF as a therapeutic target. Expert Opin Ther Targets (2010) 14(3):253–64. 10.1517/14728220903551304 20148714

[21] Lubetsky JB Dios A Han J Aljabari B Ruzsicska B Mitchell R The tautomerase active site of macrophage migration inhibitory factor is a potential target for discovery of novel anti-inflammatory agents. J Biol Chem (2002) 277(28):24976–82. 10.1074/jbc.M203220200 11997397

[22] Ouertatani-Sakouhi H El-Turk F Fauvet B Cho MK Pinar Karpinar D Le Roy D Identification and characterization of novel classes of macrophage migration inhibitory factor (MIF) inhibitors with distinct mechanisms of action. J Biol Chem (2010) 285(34):26581–98. 10.1074/jbc.M110.113951 20516071 PMC2924096

[23] Ouertatani-Sakouhi H El-Turk F Fauvet B Roger T Le Roy D Karpinar DP A new class of isothiocyanate-based irreversible inhibitors of macrophage migration inhibitory factor. Biochemistry (2009) 48(41):9858–70. 10.1021/bi900957e 19737008 PMC3607106

[24] Kerschbaumer RJ Rieger M Volkel D Le Roy D Roger T Garbaraviciene J Neutralization of macrophage migration inhibitory factor (MIF) by fully human antibodies correlates with their specificity for the beta-sheet structure of MIF. J Biol Chem (2012) 287(10):7446–55. 10.1074/jbc.M111.329664 22238348 PMC3293543

[25] Kobayashi S Satomura K Levsky JM Sreenath T Wistow GJ Semba I Expression pattern of macrophage migration inhibitory factor during embryogenesis. Mech Dev (1999) 84(1–2):153–6. 10.1016/S0925-4773(99)00057-X 10473131

[26] Faria MR Hoshida MS Ferro EA Ietta F Paulesu L Bevilacqua E. Spatiotemporal patterns of macrophage migration inhibitory factor (Mif) expression in the mouse placenta. Reprod Biol Endocrinol (2010) 8:95. 10.1186/1477-7827-8-95 20684790 PMC2922212

[27] Kevill KA Bhandari V Kettunen M Leng L Fan J Mizue Y A role for macrophage migration inhibitory factor in the neonatal respiratory distress syndrome. J Immunol (2008) 180(1):601–8. 10.4049/jimmunol.180.1.601 18097062

[28] Ietta F Todros T Ticconi C Piccoli E Zicari A Piccione E Macrophage migration inhibitory factor in human pregnancy and labor. Am J Reprod Immunol (2002) 48(6):404–9. 10.1034/j.1600-0897.2002.01152.x 12607777

[29] Chaiworapongsa T Romero R Espinoza J Kim YM Edwin S Bujold E Macrophage migration inhibitory factor in patients with preterm parturition and microbial invasion of the amniotic cavity. J Matern Fetal Neonatal Med (2005) 18(6):405–16. 10.1080/14767050500361703 16390807 PMC1383603

[30] Prencipe G Auriti C Inglese R Devito R Ronchetti MP Seganti G A polymorphism in the macrophage migration inhibitory factor promoter is associated with bronchopulmonary dysplasia. Pediatr Res (2011) 69(2):142–7. 10.1203/PDR.0b013e3182042496 21045753

[31] Thomas W Seidenspinner S Kawczynska-Leda N Kramer BW Chmielnicka-Kopaczyk M Marx A Systemic fetal inflammation and reduced concentrations of macrophage migration inhibitory factor in tracheobronchial aspirate fluid of extremely premature infants. Am J Obstet Gynecol (2008) 198(1):e1–6. 10.1016/j.ajog.2007.06.010 18166309

[32] Schlapbach LJ Graf R Woerner A Fontana M Zimmermann-Baer U Glauser D Pancreatic stone protein as a novel marker for neonatal sepsis. Intensive Care Med (2013) 39(4):754–63. 10.1007/s00134-012-2798-3 23296629

[33] Roger T Schneider A Weier M Sweep FC Le Roy D Bernhagen J High expression levels of macrophage migration inhibitory factor sustain the innate immune responses of neonates. Proc Natl Acad Sci U S A (2016) 113(8):E997–1005. 10.1073/pnas.1514018113 26858459 PMC4776487

[34] Merk M Mitchell RA Endres S Bucala R. d-dopachrome tautomerase (d-DT or MIF-2): doubling the MIF cytokine family. Cytokine (2012) 59(1):10–7. 10.1016/j.cyto.2012.03.014 22507380 PMC3367028

[35] Merk M Zierow S Leng L Das R Du X Schulte W The d-dopachrome tautomerase (DDT) gene product is a cytokine and functional homolog of macrophage migration inhibitory factor (MIF). Proc Natl Acad Sci U S A (2011) 108(34):E577–85. 10.1073/pnas.1102941108 21817065 PMC3161582

[36] Kim BS Stoppe C Grieb G Leng L Sauler M Assis D The clinical significance of the MIF homolog d-dopachrome tautomerase (MIF-2) and its circulating receptor (sCD74) in burn. Burns (2016) 42(6):1265–76. 10.1016/j.burns.2016.02.005 27209369 PMC5010466

[37] Stoppe C Rex S Goetzenich A Kraemer S Emontzpohl C Soppert J Interaction of MIF family proteins in myocardial ischemia/reperfusion damage and their influence on clinical outcome of cardiac surgery patients. Antioxid Redox Signal (2015) 23(11):865–79. 10.1089/ars.2014.6243 26234719 PMC4615780

[38] Dimova T Brouwer M Gosselin F Tassignon J Leo O Donner C Effector Vgamma9Vdelta2 T cells dominate the human fetal gammadelta T-cell repertoire. Proc Natl Acad Sci U S A (2015) 112(6):E556–65. 10.1073/pnas.1412058112 25617367 PMC4330771

[39] Schlapbach LJ Giannoni E Wellmann S Stocker M Ammann RA Graf R. Normal values for pancreatic stone protein in different age groups. BMC Anesthesiol (2015) 15:168. 10.1186/s12871-015-0149-y 26588901 PMC4654823

[40] Grass B Baumann P Arlettaz R Fouzas S Meyer P Spanaus K Cardiovascular biomarkers pro-atrial natriuretic peptide and pro-endothelin-1 to monitor ductus arteriosus evolution in very preterm infants. Early Hum Dev (2014) 90(6):293–8. 10.1016/j.earlhumdev.2014.03.002 24661445

[41] Radstake TR Sweep FC Welsing P Franke B Vermeulen SH Geurts-Moespot A Correlation of rheumatoid arthritis severity with the genetic functional variants and circulating levels of macrophage migration inhibitory factor. Arthritis Rheum (2005) 52(10):3020–9. 10.1002/art.21285 16200611

[42] Giannoni E Guignard L Knaup Reymond M Perreau M Roth-Kleiner M Calandra T Estradiol and progesterone strongly inhibit the innate immune response of mononuclear cells in newborns. Infect Immun (2011) 79(7):2690–8. 10.1128/IAI.00076-11 21518785 PMC3191988

[43] Stoll BJ Hansen NI Bell EF Walsh MC Carlo WA Shankaran S Trends in care practices, morbidity, and mortality of extremely preterm neonates, 1993-2012. JAMA (2015) 314(10):1039–51. 10.1001/jama.2015.10244 26348753 PMC4787615

[44] Pepe GJ Albrecht ED. Actions of placental and fetal adrenal steroid hormones in primate pregnancy. Endocr Rev (1995) 16(5):608–48. 10.1210/er.16.5.608 8529574

[45] Pasupuleti V Du W Gupta Y Yeh IJ Montano M Magi-Galuzzi C Dysregulated d-dopachrome tautomerase, a hypoxia-inducible factor-dependent gene, cooperates with macrophage migration inhibitory factor in renal tumorigenesis. J Biol Chem (2014) 289(6):3713–23. 10.1074/jbc.M113.500694 24356968 PMC3916569

[46] Baugh JA Gantier M Li L Byrne A Buckley A Donnelly SC. Dual regulation of macrophage migration inhibitory factor (MIF) expression in hypoxia by CREB and HIF-1. Biochem Biophys Res Commun (2006) 347(4):895–903. 10.1016/j.bbrc.2006.06.148 16854377

[47] Park AM Sanders TA Maltepe E. Hypoxia-inducible factor (HIF) and HIF-stabilizing agents in neonatal care. Semin Fetal Neonatal Med (2010) 15(4):196–202. 10.1016/j.siny.2010.05.006 20599462 PMC2924157

[48] Oda S Oda T Nishi K Takabuchi S Wakamatsu T Tanaka T Macrophage migration inhibitory factor activates hypoxia-inducible factor in a p53-dependent manner. PLoS One (2008) 3(5):e2215. 10.1371/journal.pone.0002215 18493321 PMC2375051

[49] Ren Y Tsui HT Poon RT Ng IO Li Z Chen Y Macrophage migration inhibitory factor: roles in regulating tumor cell migration and expression of angiogenic factors in hepatocellular carcinoma. Int J Cancer (2003) 107(1):22–9. 10.1002/ijc.11287 12925952

[50] Ren Y Chan HM Li Z Lin C Nicholls J Chen CF Upregulation of macrophage migration inhibitory factor contributes to induced N-Myc expression by the activation of ERK signaling pathway and increased expression of interleukin-8 and VEGF in neuroblastoma. Oncogene (2004) 23(23):4146–54. 10.1038/sj.onc.1207490 15064733

[51] Prabhu SB Rathore DK Nair D Chaudhary A Raza S Kanodia P Comparison of human neonatal and adult blood leukocyte subset composition phenotypes. PLoS One (2016) 11(9):e0162242. 10.1371/journal.pone.0162242 27610624 PMC5017693

[52] Dowling DJ Levy O Ontogeny of early life immunity. Trends Immunol (2014) 35(7):299–310. 10.1016/j.it.2014.04.007 24880460 PMC4109609

[53] Schlapbach LJ Frey S Roger T Calandra T Nelle M Aebi C Umbilical venous concentrations of estradiol in infants with early-onset neonatal sepsis and chorioamnionitis. J Neonatal Perinatal Med (2011) 4:147–54.

[54] Levy O Coughlin M Cronstein BN Roy RM Desai A Wessels MR. The adenosine system selectively inhibits TLR-mediated TNF-alpha production in the human newborn. J Immunol (2006) 177(3):1956–66. 10.4049/jimmunol.177.3.1956 16849509 PMC2881468

[55] Belderbos ME Levy O Stalpers F Kimpen JL Meyaard L Bont L. Neonatal plasma polarizes TLR4-mediated cytokine responses towards low IL-12p70 and high IL-10 production via distinct factors. PLoS One (2012) 7(3):e33419. 10.1371/journal.pone.0033419 22442690 PMC3307729

[56] Kan B Razzaghian HR Lavoie PM. An immunological perspective on neonatal sepsis. Trends Mol Med (2016) 22(4):290–302. 10.1016/j.molmed.2016.02.001 26993220 PMC5104533

[57] Shane AL Stoll BJ. Neonatal sepsis: progress towards improved outcomes. J Infect (2014) 68(Suppl 1):S24–32. 10.1016/j.jinf.2013.09.011 24140138

[58] Giannoni E Berger C Stocker M Agyeman P Posfay-Barbe KM Heininger U Incidence and outcome of group B streptococcal sepsis in infants in Switzerland. Pediatr Infect Dis J (2016) 35(2):222–4. 10.1097/INF.0000000000000974 26535881

[59] Sun H Choo-Wing R Sureshbabu A Fan J Leng L Yu S A critical regulatory role for macrophage migration inhibitory factor in hyperoxia-induced injury in the developing murine lung. PLoS One (2013) 8(4):e60560. 10.1371/journal.pone.0060560 23637753 PMC3639272

[60] Kobold S Merk M Hofer L Peters P Bucala R Endres S. The macrophage migration inhibitory factor (MIF)-homologue d-dopachrome tautomerase is a therapeutic target in a murine melanoma model. Oncotarget (2014) 5(1):103–7. 10.18632/oncotarget.1560 24406307 PMC3960192

[61] Brock SE Rendon BE Xin D Yaddanapudi K Mitchell RA MIF family members cooperatively inhibit p53 expression and activity. PLoS One (2014) 9(6):e99795 10.1371/journal.pone.0099795 24932684 PMC4059697

[62] Rajasekaran D Zierow S Syed M Bucala R Bhandari V Lolis EJ. Targeting distinct tautomerase sites of d-DT and MIF with a single molecule for inhibition of neutrophil lung recruitment. FASEB J (2014) 28(11):4961–71. 10.1096/fj.14-256636 25016026 PMC4200328

[63] Gibbons D Fleming P Virasami A Michel ML Sebire NJ Costeloe K Interleukin-8 (CXCL8) production is a signatory T cell effector function of human newborn infants. Nat Med (2014) 20(10):1206–10. 10.1038/nm.3670 25242415

[64] Lue H Dewor M Leng L Bucala R Bernhagen J. Activation of the JNK signalling pathway by macrophage migration inhibitory factor (MIF) and dependence on CXCR4 and CD74. Cell Signal (2011) 23(1):135–44. 10.1016/j.cellsig.2010.08.013 20807568 PMC3586206

[65] Yende S Angus DC Kong L Kellum JA Weissfeld L Ferrell R The influence of macrophage migration inhibitory factor gene polymorphisms on outcome from community-acquired pneumonia. FASEB J (2009) 23(8):2403–11. 10.1096/fj.09-129445 19346297 PMC2717777

[66] Awandare GA Martinson JJ Were T Ouma C Davenport GC Ong’echa JM MIF (macrophage migration inhibitory factor) promoter polymorphisms and susceptibility to severe malarial anemia. J Infect Dis (2009) 200(4):629–37. 10.1086/600894 19591577 PMC3607439

[67] Asgari S McLaren PJ Peake J Wong M Wong R Bartha I Exome sequencing reveals primary immunodeficiencies in children with community-acquired *Pseudomonas aeruginosa* sepsis. Front Immunol (2016) 7:357 10.3389/fimmu.2016.00357 27703454 PMC5028722

