# Supplementary material for: Plasma Levels of Macrophage Migration Inhibitory Factor and d-Dopachrome Tautomerase Show a Highly Specific Profile in Early Life
Source: Front Immunol. 2017 Jan 25;8:26. doi: 10.3389/fimmu.2017.00026 (PMC5263165; doi:10.3389/fimmu.2017.00026)
Supplement: Supplementary file 1 [file Table_1.DOCX]

**Supplementary Table 1**

**Surgical procedures performed in infants and children from group 3**

|  | **N = 90** |
| --- | --- |
| Adenoidectomy and/or tympanic paracentesis | 23 |
| Circumcision | 17 |
| Inguinal hernia repair | 11 |
| Removal of osteosynthesis material | 9 |
| Fracture reduction | 4 |
| Orchidopexy | 3 |
| Otoplasty | 3 |
| Tympanostomy tube removal | 3 |
| Dental surgery | 2 |
| Hydrocele repair | 2 |
| Wound care | 2 |
| Other ear, nose and throat, ophtalmologic, urologic or orthopedic procedures | 11 |
